# Genetic Modifiers of Sickle Cell Anemia Phenotype in a Cohort of Angolan Children

**DOI:** 10.3390/genes15040469

**Published:** 2024-04-08

**Authors:** Catarina Ginete, Mariana Delgadinho, Brígida Santos, Armandina Miranda, Carina Silva, Paulo Guerreiro, Emile R. Chimusa, Miguel Brito

**Affiliations:** 1 H&TRC-Health & Technology Research Center, ESTeSL-Escola Superior de Tecnologia da Saúde, Instituto Politécnico de Lisboa, 1990-096 Lisbon, Portugal; catarina.ginete@estesl.ipl.pt (C.G.); mariana.delgadinho@estesl.ipl.pt (M.D.); carina.silva@estesl.ipl.pt (C.S.); paulo.guerreiro@estesl.ipl.pt (P.G.); 2 Centro de Investigação em Saúde de Angola (CISA), Bengo 9999, Angola; santosbrigida@yahoo.com.br; 3 Hospital Pediátrico David Bernardino (HPDB), Luanda 3067, Angola; 4 Instituto Nacional de Saúde Doutor Ricardo Jorge (INSA), 1649-016 Lisbon, Portugal; armandina.miranda@insa.min-saude.pt; 5 Centro de Estatística e Aplicações, Universidade de Lisboa, 1649-013 Lisbon, Portugal; 6 Department of Applied Sciences, Faculty of Health and Life Sciences, Northumbria University, Newcastle upon Tyne NE1 8ST, UK; emile.chimusa@nothumbria.ac.uk

**Keywords:** sickle cell anemia, next generation sequencing (NGS)

## Abstract

The aim of this study was to identify genetic markers in the *HBB* Cluster; *HBS1L-MYB* intergenic region; and *BCL11A*, *KLF1*, *FOX3*, and *ZBTB7A* genes associated with the heterogeneous phenotypes of Sickle Cell Anemia (SCA) using next-generation sequencing, as well as to assess their influence and prevalence in an Angolan population. Hematological, biochemical, and clinical data were considered to determine patients’ severity phenotypes. Samples from 192 patients were sequenced, and 5,019,378 variants of high quality were registered. A catalog of candidate modifier genes that clustered in pathophysiological pathways important for SCA was generated, and candidate genes associated with increasing vaso-occlusive crises (VOC) and with lower fetal hemoglobin (HbF) were identified. These data support the polygenic view of the genetic architecture of SCA phenotypic variability. Two single nucleotide polymorphisms in the intronic region of 2q16.1, harboring the *BCL11A* gene, are genome-wide and significantly associated with decreasing HbF. A set of variants was identified to nominally be associated with increasing VOC and are potential genetic modifiers harboring phenotypic variation among patients. To the best of our knowledge, this is the first investigation of clinical variation in SCA in Angola using a well-customized and targeted sequencing approach.

## 1. Introduction

Sickle Cell Disease (SCD) is a group of inherited diseases where a single nucleotide substitution in the gene *HBB* causes an amino acid substitution from glutamic acid to valine in the β-globin subunit. This substitution affects hemoglobin behavior, forming polymers under deoxygenated conditions [1], and patients are predisposed to vaso-occlusion, ischemia, hemolysis, and inflammation [2]. It is estimated that worldwide, each year, 300,000 babies are born with SCD, with more than three-fourths of these cases being reported in Sub-Saharan Africa [3].

The most common and severe form of SCD is Sickle Cell Anemia (SCA), where two βS alleles are present. Chronic Hemolytic anemia, frequent painful crises, and extensive organ damage are common in these patients, although they tend to present very heterogeneous phenotypes with different levels of severity and life expectancy [1].

Fetal hemoglobin (HbF) is an important modulator of the SCA phenotype, having an impact on the clinical and hematological features of this disease, as high levels of HbF reduce the mean corpuscular HbS concentration and inhibit copolymerization between hemoglobin tetramers [4,5]. HbF is the most prevalent hemoglobin in the last two trimesters of gestation in humans, although some individuals continue to produce measurable amounts in adulthood. Persistent high concentrations of HbF improve overall survival and lower the number of painful crises, acute chest syndromes, and osteonecroses [5]. The variation between individuals in the regulation of HbF levels seems to be caused, in 60–90% of cases, by heritability. In approximately 50% of the cases, it is caused by single nucleotide polymorphisms (SNP) in *BCL11A*, *HBS1L-MYB*, and *HBB* [4,5,6].

Genotype–phenotype association studies are a necessity to identify new genetic markers and modifiers, better understand the different levels of severity, better establish prognosis, and even identify new potential drug targets in an era where we all intend to pursue personalized medicine. The aim of this study was to identify genetic markers associated with the heterogeneous phenotypes of SCA and assess their influence and prevalence in an Angolan population. In that regard, the *HBB* Cluster; *HBS1L-MYB* intergenic region; and *BCL11A*, *KLF1*, *FOX3*, and *ZBTB7A* genes were sequenced by NGS from samples from 192 Angolan SCA children. Moreover, we intend to compare our results with published sequences from other African populations with SCD.

## 2. Materials and Methods

### 2.1. Assessment of Hematological and Biochemical Parameters

Blood samples from 192 Angolan SCA children naïve to Hydroxyurea were collected from a cohort of Hospital Pediátrico David Bernardino and Centro de Investigação em Saúde de Angola at Hospital Geral do Bengo during routine follow-up appointments. The hematological parameters measured were complete blood count, hemoglobin, mean corpuscular volume (MCV), and mean corpuscular hemoglobin (MCH) using the XT-2000i Hematology Analyzer (Sysmex Corporation, Kobe, Japan). The hemoglobin fractions were quantified by High-Performance Liquid Chromatography (HPLC) (Biorad Variant II, Hercules, CA, USA). Biochemical blood tests included Lactate dehydrogenase (LDH), Aspartate Aminotransferase (AST), urea, creatinine, total and direct bilirubin, and Alanine Aminotransferase (ALT) using Mindray BA-88A (Mindray, Shenzhen, China) and Cobas C111 (Roche Diagnostics, Rotkreuz, Switzerland).

### 2.2. Sample Characterization

The data were analyzed according to two phenotype groups stratification. Children with previous stroke and mean LDH > 664U/L (measured in three different routine appointments) were classified as having the Hemolytic phenotype (n = 21, mean age 6.38 ± 2.20), children with no previous stroke and previous vaso-occlusive/painful crisis were classified as having the VOC phenotype (n = 138, mean age 6.75 ± 2.54), and the remaining children were classified as having the less severe phenotype (n = 33, mean age 6.21 ± 2.55). Children with HbF ≥ 7.65% (3rd Quartil value) were included in the High-HbF phenotype (n = 48, mean age 5.90 ± 2.60), and children with HbF < 7.65% were included in the Low-HbF phenotype (n = 143, mean age 6.84 ± 2.43). Data were presented as mean (SD). The t-test was used to compare the means between two independent groups and the non-parametric Kruskal–Wallis tests were applied when comparing three separate groups. Bonferroni adjustments were used for multiple testing.

### 2.3. Targeted Sequencing

After DNA extraction using the QIAamp DNA Blood Mini Kit (Qiagen GmbH, Hilden, Germany) and quantification with Qubit™ dsDNA HS fluorometric assay (ThermoFisher Scientific Inc., Waltham, MA, USA), the samples were sequenced with a custom enrichment panel (Supplementary Table S1A). Paired-end sequencing was performed on the NextSeq550 equipment (Illumina, Inc., San Diego, CA, USA) using the NextSeq 500/550 Mid-Output kit v2 (300 cycles). Reads were aligned with the reference GRCh37/hg19 human genome.

### 2.4. Variant Calling Quality Control and Annotation

Joint variant calling was conducted using GATK and BCFTOOLS [7,8]. We applied VariantMetaCaller [9] to combine and optimize the accuracy of variant calls based on the consensus of their statistical properties and discovery. The resulting VCF files were filtered using the GATK tool “VariantFiltration”.

### 2.5. Variant Annotation and Mutation Prioritization

We annotated the resulting VCF files using ANNOVAR [10] and independently performed gene-based annotation in each final VCF dataset to determine whether SNPs cause protein-coding changes and produce a list of the affected amino acids. We obtained the population frequency and pathogenicity for each variant from 1000 Genome data, Exome Aggregation Consortium (ExAC), targeted exon datasets, and COSMIC with ANNOVAR database settings [10]. We leveraged ANNOVAR’s library and RefGene to extract gene function and different functional predictions. ANNOVAR has up to 21 different mutation score tools including SIFT, LRT, MutationTaster, MutationAssessor, FATHMM and FATHMM-MKL, RadialSVM, LR [11], PROVEAN, MetaSVM, MetaLR, CADD, GERP++, DANN, M-CAP, Eigen, GenoCanyon, Polyphen2-HVAR and HDIV, PhyloP, and SiPhy [11,12,13,14,15,16,17,18,19,20,21,22,23,24,25,26,27,28]. In addition, conservative and segmental duplication sites were included, and dbSNP code and clinical relevance were reported in dbSNP. From the resulting functional annotated dataset, we independently filtered for predicted functional status (of which each predicted functional status is “deleterious” (D), “probably damaging” (D), “disease-causing-automatic” (A), or “disease-causing” (D). We selected candidate mutation based on the following: (1) casting vote approach implemented in our custom python script, retaining only a variant if it had at least 17 predicted functional status of “D” or “A” out of 21 and (2) further filtering for rarity, exonic variants, and nonsynonymous mutations and with a high-quality call from the retained variants from step 1 above.

### 2.6. Network and Enrichment Analysis

From the obtained candidate lists of predicted mutant variants, we reconstructed their functional, physical, and co-expression-interacting network GeneMania [29]. We further examined how these genes within the constructed networks were associated with human phenotypes, pathways, biological processes, and molecular functions using Enrichr [30]. The most significant pathways enriched for genes in the networks were selected from various bioinformatics databases [30]. Gene ontology terms and annotations from the Gene Ontology databases were extracted for cellular components, biological processes, and molecular functions.

### 2.7. Principal Component Analysis (PCA)

To evaluate the extent of substructure within Angolan SCA, we leverage the curated 192-phased haplotypes dataset, which resulted from Eagle [31], to perform genetic structure analysis based on Principal Component Analysis (PCA) using smartpca [32]. Genesis software http://www.bioinf.wits.ac.za/software/genesis was used to plot PCA (accessed on 10 January 2024).

We further performed a PCA analysis to investigate the genetic structure of Angola HbF patients with other population groups. We accessed VCF files from the 1000 Genomes Project (1KGP) Consortium, 2015, and the African Genome Variation Project (AGVP), which recently characterized the admixture across 18 ethnolinguistic groups from Sub-Saharan Africa [33]. A quality control check was conducted on these VCF files using Plink [34], and we ultimately retained 2504 and 2428 samples from 1KGP and AGVP, respectively. Based on sample description (population or country labels), population ethnolinguistic information [35,36] was utilized to categorize the obtained data per ethnolinguistic cultural group as described in Supplementary Table S2, resulting in 20 ethnolinguistic cultural groups and our samples. The first 20 principal components were computed from EIGENSTRAT package via smartpca, comparing Angolan SCA and these groups; the second PCA compared SCA patients among themselves, and phylogenic trees were also plotted.

### 2.8. Distribution of Minor Allele Frequency and Gene-Specific in SNP Frequencies

The distribution of the minor allele frequency (MAF) was investigated to examine the extent of common and rare variants across 9 selected ethnic groups (KhoeSan, Niger–Congo Bantu, Niger–Congo Volta Niger, Niger–Congo West, European South, European–USA, East Asian, South Asian, and African-American) and Angola SCA patients group. Similarly, a second comparison was conducted just among Angola SCA groups, including SCA VOC, SCA Hemolytic, SCA Low and High HbF. To this end, the proportion of minor alleles was categorized into six ranges (0–0.05, >0.05–0.1, >0.1–0.2, >0.2–0.3, >0.3–0.4, >0.4–0.5) with respect to each ethnic group with a disease. The MAF per SNP for each category was computed using Plink software. Furthermore, the fraction of gene-specific SNP frequency for each gene was computed, assuming SNPs upstream and downstream within a gene region are close and possibly in Linkage Disequilibrium (LD), obtained from dbSNP database [17]. MAF per SNP was aggregated as per our previous studies [37,38].

### 2.9. Identity by Descent (IBD) and Functional Genomics

Leveraging the 192 samples of Angola SCA, we examine the overall genomic identity by descent (IBD) sharing between pairs of SCA patients, aiming to look at the genomics regions of interest or long-shared segments. After phasing the data using Eagle 2.0 [31], we inferred the segments of IBD from the Refined IBD algorithm [39]. The genomic IBD segments among the 192 Angola SCA patients were evaluated, and the shared segments between the SCA groups (VOC, Hemolytic, Low/High HbF) were compared. A cut-off of 250 kb was applied to retain segments of shared IBD, and genes were mapped to these genomic regions to examine their potential functional biological network and, in addition, their functional partners. Additional enrichment analyses were explored to gain insight into potential disease-compromised networks.

### 2.10. HbF Association Testing

HbF association testing was performed using EMMAX [40] on curated dataset as a result of genetics association quality-control guidelines. EMMAX was run to detect possible associations, and we generated a pairwise relatedness matrix from the dataset, which is representative of the structure of the samples using EMMAX-kin. Given the SNPs for association with HbF, we, therefore, used a genome-wide significance level of 0.05/m where m is the total number of tested variants.

### 2.11. Meta-Analysis of Angolan HbF and Other African Ancestry HbF

To identify associations with small effect sizes, which are not usually detected by standard genetic association methods, summary statistics from Tanzania [41] and African-Americans [42] were combined with those from our study in a single association dataset. A fixed effects model [43] based on inverse-variance weighted effect size was used to combine the log odds ratio and standard error from the combined GWAS summary statistics dataset. Random effects and binary effects models, as described in the MetaSoft program, were applied [43], and the *p*-values from fixed effect model and M-values (the posterior probability that the effect exists in the study) were used to assess the level of significance. Variants were retained to be significant for M-values > 8.5 across all the studies, and *p*-values from fixed effect were lesser and equal to 0.05/M, where M is the total number of variants tested for meta-analysis.

### 2.12. Rare-Variant Association and Burden Tests

To account for rare variants and sample size and leverage possible effects from variants not included in association test and meta-analysis above and those not meeting the genome-wide significance level, an optimal unified sequence kernel association test (SKAT-O) [44], aggregating SNP effects at gene level, was performed to discriminate quantitative traits appropriately. We utilized the linear weighted kernel within SKAT-O and set the missing cut-off to 0.9 to calculate the permutation *p*-value while adjusting for age and principal.

### 2.13. Estimating Functional Heritability from GWAS Dataset

Approaches based on association summary statistics gained critical interest in the “Omics” era due to the privacy advantages they present and, particularly, their reduction of computational cost [45,46]. We applied LDAK [47] to estimate the functional SNP-heritability of HbF from summary statistics. Briefly, we excluded the major Histocompatibility Complex (MCH) region (25,000,000–40,000,000) on chr6 and the sickle cell (HbS) region on chr11:2,500,000–6,500,000 to avoid potential biases. We constructed Genomic Relatedness Matrix (GRM) from pruned, high-quality, independent autosomal SNPs (independent pairwise 50 10.2) and obtained a list of samples with a relatedness threshold >5%. We then computed GRMs using all SNPs for each cohort and excluded one of any pair of samples with relatedness threshold >5%, and the functional enrichment and SNP-heritability were estimated as recommended [48]

## 3. Results

### 3.1. Participant Characteristics and Targeted Variant Discovery

The study sample consisted of 192 SCA Angolan children (99 female), aged between 3 and 12 years old (mean (SD): 6.6 (2.5)). The percentage of HbF ranged between 0.7 and 23.8% (5.65 (3.98)). The children were grouped according to the value of HbF (Low HbF < 7.65% and High HbF ≥ 7.65%) and according to previous manifestations/phenotype (Hemolytic, vaso-occlusive, and less severe phenotypes) (Table 1).

A total number of 5,019,378 variants (1.7% insertion, 1.9% deletion, 5.4% structural variants, 0.012% multi-nucleotide variants, and 91% SNPs) were called in the targeted sequence dataset, of which 1.3% and 54% were exonic and intergenic, respectively, and they were distributed as 0.001% stop loss, 0.02% stop gain, 0.9% synonymous, 0.56% non-synonymous, and 0.05% splice site variants in the dataset. Supplementary Figure S1 illustrates the quality control of the sequence alignment data.

### 3.2. In Silico Mutational Burden of Genes in Participants

**Figure 1:**
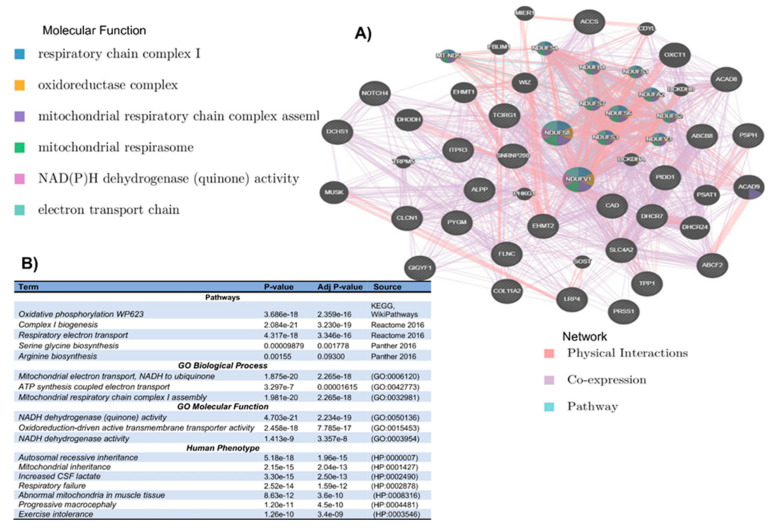
(**A**) Physical, co-expression, and functional networks of the 26 genes where significant differences in the burden of non-synonymous, function-altering variants were identified among the 192 SCA patients. (**B**) Pathways associated with the 26 genes where significant differences in the burden of non-synonymous, function-altering variants were identified among the 192 SCA.

To examine potential genetic modifiers, we performed mutation prioritization and examined the in silico biological functional pathways’ relationship to these mutations through reconstructing their physical, functional, and co-expression networks as well as enrichment analysis. Among 192 SCA patients, we detected significant differences in the burden of non-synonymous, function-altering variants in a total of 26 genes (Supplementary Table S1B,C) ranging in chromosome 11: p11.2, p15.4, p15.5, q13.1, q13.2, q13.4, and q25; chromosome 2: p23.3, q11.2, and q37.1; chromosome 6: p21.31, p21.32, and p21.33; and chromosome 7: p11.2, q22.1, q32.1, q34, and q36.1. The physical, co-expression, and functional networks of these genes (Figure 1A) are enriched with pathways (Figure 1B) such as Oxidative phosphorylation (*p* = 2.359 × 10^−16^), Respiratory electron transport (*p* = 3.346 × 10^−16^), and Arginine biosynthesis (*p* = 0.009). These pathways point to relevant pathophysiological mechanisms, including some that are already therapeutic targets.

Our findings from the rare variant-based gene-burden association tests (Table 2) included most of the variants found to harbor recurrent deleterious variants (Supplementary Table S1C) targeting, in LD, several variants from the study’s targeted regions.

### 3.3. Population Structure and Distribution of Gene-Specific in SNP Frequencies

HbF samples from Angola were merged with a combined 4932 samples from 1KGP [49] and the AGVP [33], resulting in 237,572 common variants from the study’s targeted sequence data. Based on sample description population and country labels, these 4932 samples were grouped (Supplementary Table S2) based on culture and ethnolinguistic information [35,36], resulting in 20 worldwide ethnolinguistic cultural groups (WECG).

**Figure 2:**
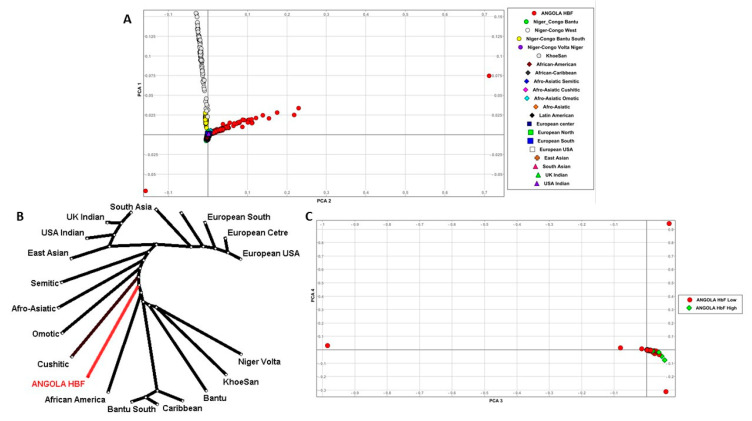
Principal Component Analysis (PCA) of Sickle Cell Disease cohorts from Cameroon and Tanzania. (**A**) PCA plot of the first and the second eigenvectors for 20 ethnic groups with SCD from Cameroon and Tanzania. (**B**) Phylogeny tree showing evolutionary partnership between SCD cohorts and general populations from 20 ethnic groups. (**C**) PCA plot of only Africa-specific ethnicities with SCD cohorts in the first and the second eigenvectors.

PCA based on these 237,572 common variants showed that the study samples clustered separately from the rest of these 20 WECG (Figure 2). It particularly formed a clearly distinct cluster from the Khoisan group. PCA plots (Supplementary Figure S2 and Figure 2) showed no global population differences among the SCA patients, i.e., Hemolytic and VOC patients clustered together, except for three patients with VOC-independent outliers. Supplementary Table S3 illustrates the genetics distance (FST) among the 20 WECG and SCA Angola.

**Figure 3:**
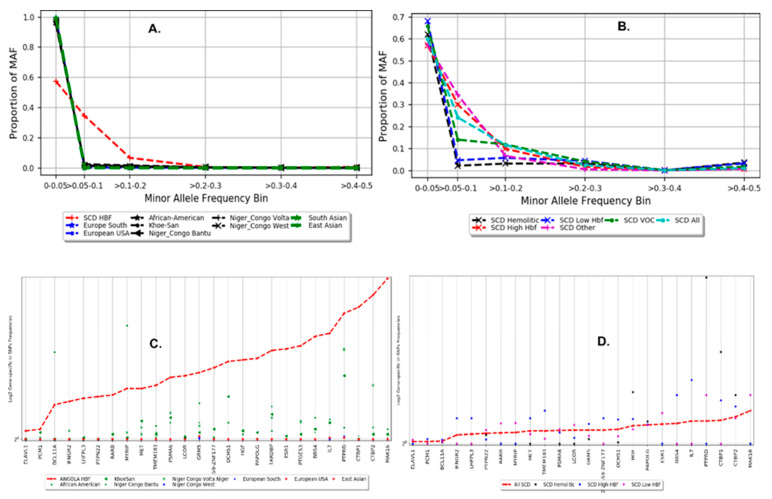
(**A**,**B**) The distribution of the minor allele frequency categorized into 6 ranges (0–0.05, >0.05–0.1, >0.1–0.2, >0.2–0.3, >0.3–0.4, >0.4–0.5) with respect to each ethnolinguistic cultural group regarding SNPs associated with (**A**) HIV-, (**B**) TB-, (**C**) Malaria-, (**D**) Sickle Cell Disease-, and ACG-specific genes. C-D gene-specific SNP minor allele frequency: The distribution of the minor allele frequency at gene level for HIV, TB, Malaria, Sickle Cell Disease, and ACG (actionable genes) among 20 ethnolinguistic cultural groups.

We observed a variation in the distribution of minor alleles at rare variants within MAF range 0.0–0.05 and as well as at MAF range 0.1–0.2 between SCA Angola and nine selected major WECG (Figure 3A). Among SCA Angola samples, variations in the distribution of MAF were observed in SNP frequencies ranging between 5% and 20% (Figure 3B), suggesting possible mutations and genetic modifiers may result in heterogeneous phenotypes of SCA observed in our study. The substantial variation of gene-specific SNP frequencies from the selected top pathogenic genes (Supplementary Table S1B) was observed within SCA Angolan samples (Figure 3D) and between Angolan and the selected nine WECG (Figure 3C). This may support the hypothesis that genetics modifiers may result in potential clinical variability of SCA phenotypes.

### 3.4. Association and Meta-Analysis

**Figure 4:**
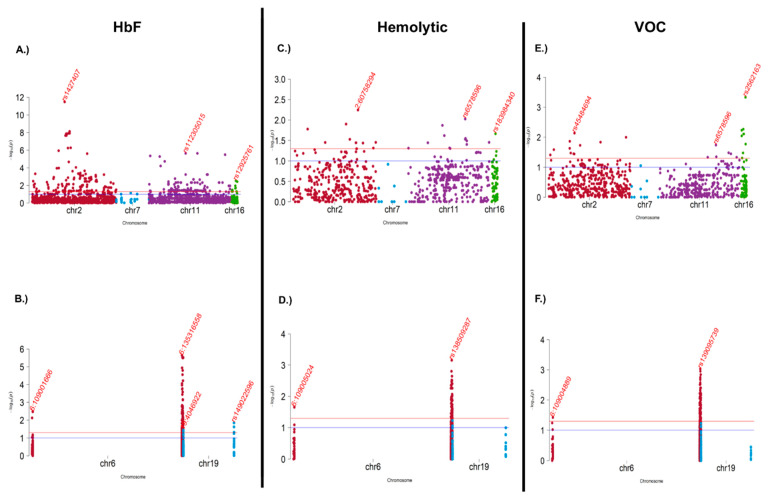
Genome-wide association analysis of unimputed and imputed SCD genotype data. (**A**,**B**) Manhattan plot of the GWAS association test of both unimputed Cameroon SCD discovery and replication cohorts. (**C**,**D**) Manhattan plot of the GWAS association test of imputed combined Tanzania and Cameroon SCD cohorts. (**E**,**F**) Manhattan plot of the GWAS association test of imputed Cameroon SCD replication cohort. Red line denotes the genome-wide significance thresholds. Blue line denotes the level of suggestive significance. The insert in each Manhattan plot is Quantile–Quantile (Q-Q) plot of expected vs. observed -log10P value within the genomic inflation factor (lambda GC).

We analyzed data from 192 quantitative HbF based on variants discovered from the study’s targeted sequence data. As expected, we did not observe a substantial population substructure, and following data quality control, three sample outliers were removed. To account for both population stratification and hidden relatedness, we applied the mixed model approach EMMAX [40]. The Q-Q plots of genomic control factor effects shown in Figure 4A are acceptable (λGC = 1.04) and suggest little departure from the null expectation, except at the right end tail of the distribution. As shown in Table 3 and Figure 4A, two SNPs in the intronic region of chromosome 2q16.1, rs1427407 (*p* = 1.29 × 10^−09^, MAF = 0.22), and rs71327644 (*p* = 7.39 × 10^−08^, MAF = 0.30) are genome-wide and significantly associated with decreasing HbF. These SNPs are associated with the *BCL11A* gene. Previous studies showed that the γ-globin repressor *BCL11A* is a target for the development of therapies for β-hemoglobinopathies by reactivating HbF. *BCL11A* interacts with 43 genes (Supplementary Figure S3A) either in physical, co-expression, or both pathway networks. Importantly, through the cross-HbF meta-analysis of Angola, Tanzania, and West Africa, we replicated the chromosome region 2p16.1 of *BCL11A*, and the meta-analysis fixed effect test enabled the recovery of five several variants near *BCL11A* within 2p16.1 harboring another five genes, including *IFITM3P9, RPL26P13, RNU6-612P, ATP1B3P1*, and *PAPOLG* (Table 4).

We additionally performed a VOC versus Hemolytic logistic association test, and no variant reached the genome-wide level of significance; however, several variants reached a nominal level of significance (<0.05) at 18 chromosomal regions (Figure 4C, Supplementary Table S4(1)), including chromosome 2 (p11.1, p24.1, q14.3, and q24.2), chromosome 6 (p22.3, p24.2, q11.1, q16.1, and q21), chromosome 7 (p15.3, q11.21, and q21.11), chromosome 11 (p11.12, q11, and q13.2), and chromosome 16 (p11.2, p12.3, and q11.2). These identified variants within the chromosomal regions are nominally associated with increasing VOC (OR > 1, Supplementary Table S4(1)) and are potential genetic modifiers inducing phenotypic variation among patients with VOC and Hemolytic phenotypes in Angola SCA. Conversely, we conducted a lower versus higher HbF logistic association test, and no variants, at genome-wide level of significance, were detected. However, variants in Figure 4D and Supplementary Table S4(2) ranging in 12 chromosomal regions were nominally associated with lower HbF (increasing the lowness of HbF, OR > 1, Supplementary Table S4(2)), including chromosome 2 (2p11.1, 2q24.2, 2p16.1, and q32.2), chromosome 16 (p11.2, q11.2, and q23.2), chromosome 6 (p22.1 and q16.3), chromosome 11 (q11 and q24.3), and chromosome 7q11.21. Most of the genes associated with these nominally significant variants, including *BCL11A* (Supplementary Table S4(2)), are, interestingly, part of the *BCL11A* functional/physical and co-expression network.

## 4. Discussion

The phenotype heterogeneity of SCA presents a challenge for patients’ clinical management. Our study addresses the issue of potential function-altering variants and genetic modifiers of variation associated with these heterogeneous phenotypes. We utilized a design that ascertained HbF individuals from the extremes of genetic risk, including Hemolytic and VOC phenotypes. With this, we were able to generate a targeted sequence catalog of 192 Angolan samples from high-quality variants, calling on 5,019,378 variants with high confidence. An SCD-specific population structure study was conducted within our population samples and between the 20 WECG, which showed that the study samples clustered separately from the rest of these groups (Figure 2) and, particularly, formed a clearly distinct cluster from the Khoisan group, an ethnic group from southern African with fewer incidences of Malaria and SCA, which is not surprising because samples from Angola were not included in the 1KGP. Additionally, we observed variation in the distribution of minor alleles at rare variants within the MAF range of 0.0–0.05, as well as at the MAF range of 0.1–0.2 between SCA Angola and WECG (Figure 3A). Within the SCA Angolan samples, variation in the distribution of MAF was observed in SNP frequencies ranging between 5% and 20% (Figure 3B), suggesting possible mutations and genetic modifiers may result in heterogeneous phenotypes of SCA.

The first key finding points to significant differences in the burden of non-synonymous, function-altering variants in a total of 26 genes (Supplementary Table S1B,C), of which a strong variation in gene-specific SNPs was observed within SCA Angolan samples (Figure 3D), as well as between Angolan and WECG (Figure 3C), supporting the hypothesis that genetic modifiers may result in a potential clinical variability in SCA phenotypes. These genes are enriched for deleterious and loss-of-function mutations in phenotypically defined groups of Angolan SCA patients and with evidence of genetic association with different phenotypes, providing support for the polygenic view of the genetic architecture of SCD phenotypic variability.

Notably, pathways (Figure 1A,B), including Oxidative phosphorylation, Respiratory electron transport, and Arginine biosynthesis pathways represented by these 26 genes, point to relevant pathophysiological mechanisms and are already therapeutic targets [37,50]. Importantly, Arginine biosynthesis is a key factor in the hemolysis–endothelial dysfunction observed in SCD and has become a target for therapeutic interventions [37,50]. This finding is novel and noteworthy and will contribute to a greater understanding of the variability in the clinical expression of SCA, and our identified genes and pathways suggest new avenues for other interventions.

The second key finding of this paper suggests two SNPs in the intronic region of 2q16.1, harboring the genome-wide *BCL11A* gene, which is significantly associated with decreasing HbF. Interestingly, through the cross-HbF meta-analysis of Angola, Tanzania, and West Africa, we replicate the chromosome region 2p16.1 of *BCL11A*, and the meta-analysis fixed effect test enabled the recovery of several variants near *BCL11A* within 2p16.1, as well as other five genes, including *IFITM3P9* (processed pseudogene), RPL26P13 (processed pseudogene), *RNU6-612P* (snRNA), *ATP1B3P1* (processed pseudogene), *PAPOLG* (protein coding). *BCL11A* is a potent silencer of fetal hemoglobin and controls the β-globin gene cluster in concert with other factors. Our study demonstrated that *BCL11A* interacts with 43 genes (Supplementary FigureS S3A and S4) either in physical, co-expression, or both pathway networks. This network is enriched in the B Cell Receptor Signaling pathway and associated with the Gastrointestinal stroma tumor (HP:0100723) human phenotype (Supplementary Figure S3B).

Our study leveraged HbF association summary statistics based on targeted sequence to partition the cumulative heritability into 65 different functional categories and biological pathways. We observed cumulative heritability in fewer categories, such as in fetal DNase I hypersensitive site and lysine H3K27 acetylation (Supplementary Figure S4), supporting the polygenic view of the genetic architecture of HbF SCD and demonstrating consistency with the hypothesis that the vast proportion of complex, heritable traits/diseases is explained by SNPs with small effect sizes.

Furthermore, this study identified a set of variants in 18 chromosomal regions (Figure 4B) to nominally be associated with increasing VOC (Supplementary Table S4(1)). This study also found that these variants are potential genetic modifiers causing phenotypic variation among patients with VOC and Hemolytic phenotypes in Angola SCA. This study additionally detected a set of variants ranging in 12 chromosomal regions to nominally be associated with lower HbF (Supplementary Table S4(2)). Most of the genes associated with these nominally significant variants, including *BCL11A*, are interestingly part of the *BCL11A* functional/physical and co-expression network (Supplementary Figure S3A).

To our knowledge, this is the first investigation of clinical variation in SCA in Angola using a well-customized and targeted sequencing approach. The strengths of the study include well-defined clinical groups, sites where treatment is unlikely to confound outcomes, the use of several different but complementary analytical approaches, and the linking of the identified genes and pathways to published therapeutic and transcriptomic data. Nonetheless, the study has some limitations; some of our findings may depend greatly on laboratory experiments, and the distribution of actionable genes across SCA phenotypic groups may depend on continuous genetic diversity, natural selection, and genetic drift. Such a study paves the way for the continuous analysis of SCA-specific actionable and therapeutic genes and their genetic mechanism underpinning SCA.

In summary, we reported a well-customized and targeted sequence catalog of 192 Angolan samples from high-quality variants, more specifically, 5,019,378 high-confidence variants. We generated a catalog of candidate modifier genes that clustered in pathophysiological pathways important for SCA, supporting the polygenic view of the genetic architecture of SCD phenotypic variability with implications for therapeutic intervention. We also identified and replicated the association of *BCL11A* in decreasing HbF and constructed a physical, co-expression pathway network for *BCL11A*, harboring 43 other genes. Moreover, we generated a catalog of nominally significant candidate genes associated with increasing VOC and a set of nominally significant candidate genes associated with lower HbF. This study fills an important knowledge gap by using a precise panel in a targeted sequencing approach focusing on deleterious coding variants that are important in two specific phenotypic categories of SCA patients (VOC and Hemolytic). This study, thus, makes significant contributions to the present knowledge of the natural history and clinical heterogeneity of SCA, with the potential to inform the design of new therapeutic measures.

## Figures and Tables

**Table 1:** Hematological and clinical characteristics of the patients according to their phenotype and HbF groups (Low and High).

	*Phenotype*				*HbF Groups*		
	Hemolytic (n = 21)	Vaso-Oclusive (n = 138)	Less Severe (n = 33)	*p*-Value * (Kruskal–Wallis)	Low—HbF (n = 143)	High—HbF (n = 48)	*p*-Value (*t*-Test)
	Mean (SD)	Mean (SD)	Mean (SD)		Mean (SD)	Mean (SD)	
**Fetal Hemoglobin (%)**	5.11 (3.07)	5.62 (3.94)	6.14 (4.61)	0.765	3.89 (1.96)	10.92 (3.79)	-
**Hemoglobin (g/dL)**	7.02 (1.01)	7.38 (0.98)	7.34 (0.89)	0.188	7.17 (0.91)	7.81 (1.01)	**<0.001**
**Reticulocyte (%)**	11.55 (3.2)	9.94 (4.84)	11.1 (4.54)	**0.022**	10.43 (4.75)	9.98 (4.44)	0.999
**Erythrocytes (10^12^L)**	2.68 (0.45)	3 (0.65)	2.88 (0.56)	0.059	2.9 (0.61)	3.07 (0.66)	0.999
**MCV (fL)**	80.1 (6.41)	76.45 (9.21)	77.61 (7.67)	0.072	76.7 (8.83)	78.02 (8.55)	0.999
**MCH (pg)**	26.44 (2.37)	25.12 (3.07)	25.88 (2.81)	0.098	25.2 (2.96)	25.95 (3.01)	0.999
**White blood cells (10^9^L)**	13.48 (3.38)	14.07 (5)	14.12 (3.99)	0.735	14.11 (4.87)	13.77 (4.119	0.999
**Neutrophil (10^9^L)**	6.15 (2.15)	5.88 (2.34)	5.91 (2.55)	0.616	5.96 (2.31)	5.82 (2.49)	0.999
**Platelet (10^9^L)**	382.32 (122.85)	440.89 (180.74)	448.97 (152.62)	0.182	437.08 (180.36)	432.8 (143.78)	0.999
**Transfusions/year**	0.86 (0.91)	0.34 (0.47)	0.35 (0.32)	**0.009**	0.46 (0.59)	0.22 (0.31)	**<0.001**
**Hospitalizations/year**	0.9 (0.77)	0.45 (0.47)	0.37 (0.3)	**0.003**	0.54 (0.54)	0.31 (0.32)	**0.011**

**Table 2:** Significant genes from gene-set rare-variant association analyses in Angola Sickle Cell Diseases.

Gene	CHR	Start Position	End Position	Band	Gene Biotype	*p*	Nbr Marker	Marker Tested	Marker Rare	Marker Common
*LHFPL3*	chr7	104328603	104908561	q22.2	protein-coding	1.65 × 10^−5^	40	31	31	9
*ZNF559-ZNF177*	chr19	9324174	9382617	p13.2	protein-coding	0.00012	8	5	5	3
*TMEM181*	chr6	158536436	158635429	q25.3	protein-coding	0.0033	40	29	29	11
*PAPOLG*	chr2	60756253	60802086	p16.1	protein-coding	0.0035	39	33	33	6

**Table 3:** Top significant variants from the association analyses in Angola HBF Sickle Cell Diseases. The HbF shows significant association with *BCL11A* and is nominally associated with 4 other genes, including *OR4C46*, *GFOD1*, *ACTR3BP2*, and *MUC3A*.

CHR	BP	SNP	MAF	A1/A2	Gene	Band	Func	β	SE	*p*
2	60718043	rs1427407	0.22	T/G	*BCL11A*	p16.1	intronic	−3.11	0.49	1.29 × 10^−9^
2	60723096	rs71327644	0.30	C/CA	*BCL11A*	p16.1	intronic	−2.55	0.46	7.39 × 10^−8^
2	60724087	rs1896296	0.3	G/T	*BCL11A*	p16.1	intronic	−2.51	0.48	3.9 × 10^−7^
2	60724086	rs1896295	0.29	T/C	*BCL11A*	p16.1	intronic	−2.45	0.48	7.36 × 10^−7^
2	60719970	rs766432	0.3	C/A	*BCL11A*	p16.1	intronic	−2.43	0.48	9.38 × 10^−7^
2	60720589	rs10195871	0.35	A/G	*BCL11A*	p16.1	intronic	−2.43	0.48	9.38 × 10^−7^
2	60720757	rs10172646	0.32	G/A	*BCL11A*	p16.1	intronic	−2.43	0.48	9.38 × 10^−7^
2	60721347	rs7557939	0.3	G/A	*BCL11A*	p16.1	intronic	−2.43	0.48	9.38 × 10^−7^
2	60720951	rs4671393	0.3	A/G	*BCL11A*	p16.1	intronic	−2.43	0.48	9.38 × 10^−7^
2	60720318	rs34211119	0.3	gtt/gt	*BCL11A*	p16.1	intronic	−2.43	0.48	9.38 × 10^−7^
2	60721311	rs7584113	0.30	A/G	*BCL11A*	p16.1	intronic	−2.41	0.48	1.29 × 10^−6^
2	60720246	rs11886868	0.3	C/T	*BCL11A*	p16.1	intronic	−2.37	0.48	1.72 × 10^−6^
2	60719074	rs1896294	0.3	C/T	*BCL11A*	p16.1	intronic	−2.37	0.48	1.72 × 10^−6^
2	60725451	rs7606173	0.42	C/G	*BCL11A*	p16.1	intronic	1.81	0.41	2 × 10^−5^
2	60722040	rs6706648	0.38	T/C	*BCL11A*	p16.1	intronic	1.87	0.44	3.01 × 10^−5^
2	60710738	rs11692396	0.25	G/A	*BCL11A*	p16.1	intronic	−1.98	0.5	9.03 × 10^−5^
11	51572589	1032:33:00	0.06	T/G	*OR4C46*	q11	intergenic	−3.51	0.92	0.00019
2	60723108	rs45606437	0.32	A/AC	*BCL11A*	p16.1	intronic	1.61	0.43	0.00024
6	13542533	rs1195623516	0.05	T/C	*GFOD1*	p23	intergenic	−3.59	0.97	0.00029
6	13542532	rs754078005	0.051	A/G	*GFOD1*	p23	intergenic	−3.59	0.97	0.00029
2	92312693	rs201391728	0.25	G/T	*ACTR3BP2*	p11.1	intergenic	−1.97	0.55	0.00045
2	92307971	rs201915260	0.18	G/T	*ACTR3BP2*	p11.1	intergenic	2.02	0.58	0.00057
7	100550995	rs1394766104	0.088	G/A	*MUC3A*	q22.1	exonic	2.64	0.78	0.00081
2	92312692	rs200577446	0.22	G/T	*ACTR3BP2*	p11.1	intergenic	−1.94	0.58	0.0009

**Table 4:** Cross-meta-analysis of Sickle Cell Disease cohorts: Angola, Tanzania, and West Africa. Cross-Sickle Cell Disease studies meta-analysis: African and Africa-American. The cross-meta-analysis shows a significant association of HbF with several variants in chromosome region of 2p16.1 near *BCL11A*, including 5 other genes within the region 2p16.1. P1, P2, and P3 stand for Angola, Tanzania, and West Africa study P values. M1, M2, and M3 stand for posterior probabilities that the effect exists within Angola, Tanzania, and West Africa studies, respectively.

CHR	SNP	BP (hg19)	A1/A2	*p* Values FE	OR ± STD FE	Pvalues R E	OR ± STD RE	*p* Values BE	P1	P2	P3	M1	M2	M3
2	rs147630502	60718043	T/G	5.0 × 10^−29^	0.2 ± 1.1	0.002	0.2 ± 1.7	4.2 × 10^−30^	1.2 × 10^−9^	2.2 × 10^−24^	0.17	1.0	1.0	0.2
2	2:60720951	60720951	A/G	3.9 × 10^−23^	0.2 ± 1.2	0.0005	0.2 ± 1.5	1.0 × 10^−22^	9.3 × 10^−7^	5.2 × 10^−19^	0.81	1.0	1.0	0.7
2	2:60719970	60719970	C/A	7.3 × 10^−23^	0.25 ± 1.1	0.0007	0.2 ± 1.5	1.5 × 10^−22^	9.38 × 10^−7^	8.1 × 10^−19^	0.64	1.0	1.0	0.7
2	2:60720757	60720757	G/A	1.3 × 10^−19^	0.3 ± 1.13	0.02	0.3 ± 1.7	2.8 × 10^−22^	9.38 × 10^−7^	9.04 × 10^−19^	0.79	1.00	1.0	0.7
2	2:60720589	60720589	A/G	2.2 × 10^−19^	0.3 ± 1.1	0.03	0.3 ± 1.7	2.2 × 10^−22^	9.38 × 10^−7^	7.7 × 10^−19^	0.95	0.9	1.0	0.6
2	2:60719074	60719074	C/T	2.0 × 10^−18^	0.3 ± 1.14	0.09	0.3 ± 1.9	2.2 × 10^−23^	1.71 × 10^−6^	6.1 × 10^−20^	0.31	1.0	1.0	0.0
2	2:60682447	60682447	G/A	1.2 × 10^−12^	2.6 ± 1.12	0.0003	2.2 ± 1.3	1.2 × 10^−12^	0.008	1.04 × 10^−11^	0.29	0.96	1.0	0.6
2	2:60755798	60755798	T/C	1.4 × 10^−11^	3.0 ± 1.17	1.4 × 10^−11^	2.9 ± 1.2	4.1 × 10^−11^	0.008	3.02 × 10^−10^	0.84	0.97	1.0	0.7
2	2:60757130	60757130	C/A	1.8 × 10^−11^	2.9 ± 1.17	1.8 × 10^−11^	3.0 ± 1.8	6.4 × 10^−11^	0.026	2.63 × 10^−10^	0.49	0.95	1.0	0.8
2	2:60750303	60750303	T/C	4.4 × 10^−11^	2.8 ± 1.16	4.4 × 10^−11^	2.8 ± 1.2	1.7 × 10^−10^	0.009	1.6 × 10^−10^	0.32	0.96	1.0	0.8
2	2:60697654	60697654	A/C	6.7 × 10^−11^	2.4 ± 1.14	6.7 × 10^−11^	2.4 ± 1.1	1.5 × 10^−10^	0.039	3.4 × 10^−10^	0.88	0.93	1.0	0.7
2	2:60755762	60755762	T/C	7.1 × 10^−11^	2.8 ± 1.17	7.1 × 10^−11^	2.8 ± 1.2	2.0 × 10^−10^	0.011	1.2 × 10^−9^	0.84	0.97	1.0	0.7
2	2:60756755	60756755	G/C	7.4 × 10^−11^	2.8 ± 1.17	7.4 × 10^−11^	2.8 ± 1.2	2.3 × 10^−10^	0.016	1.2 × 10^−9^	0.65	0.9	1.0	0.8
2	rs575474598	60710738	G/A	9.0 × 10^−11^	0.4 ± 1.14	0.32	0.5 ± 2.2	1.7 × 10^−16^	9.03 × 10^−5^	2.4 × 10^−14^	0.01	0.9	1.0	0.0
2	rs1236323224	60756504	T/C	1.3 × 10^−10^	2.8 ± 1.17	1.3 × 10^−10^	2.8 ± 1.2	4.3 × 10^−10^	0.016	2.05 × 10^−9^	0.72	0.9	1.0	0.8
2	2:60736852	60736852	A/G	1.8 × 10^−10^	2.6 ± 1.15	7.7 × 10^−5^	2.6 ± 1.3	4.2 × 10^−10^	0.002	6.4 × 10^−9^	0.41	0.9	1.0	0.7
2	2:60743605	60743605	G/A	3.6 × 10^−10^	2.5 ± 1.15	0.005	2.4 ± 1.4	2.2 × 10^−10^	0.005	1.5 × 10^−9^	0.51	0.9	1.0	0.5
2	2:60729702	60729702	G/A	1.1 × 10^−8^	2.4 ± 1.16	0.26	1.7 ± 1.7	4.6 × 10^−9^	0.011	1.9 × 10^−8^	0.15	0.9	1.0	0.3
2	2:60684034	60684034	C/T	4.8 × 10^−7^	3.1 ± 1.25	4.8 × 10^−7^	3.1 ± 1.3	2.0 × 10^−6^	0.054	6.1 × 10^−6^	0.17	0.9	1.0	0.8
2	2:60698461	60698461	T/C	5.9 × 10^−7^	1.9 ± 1.13	0.1	1.6 ± 1.4	1.2 × 10^−7^	0.092	1.06 × 10^−7^	0.7	0.8	1.0	0.2
2	2:60708597	60708597	C/T	9.2 × 10^−7^	0.5 ± 1.13	0.4	0.6 ± 1.8	1.1 × 10^−9^	0.004	8.01 × 10^−9^	0.02	0.9	1.0	0.004
2	2:60701335	60701335	C/T	1.4 × 10^−6^	1.8 ± 1.13	0.3	1.4 ± 1.4	1.2 × 10^−7^	0.6	2.8 × 10^−8^	0.57	0.4	1.0	0.1
2	2:60698397	60698397	C/A	3.8 × 10^−6^	1.8 ± 1.13	0.6	1.3 ± 1.8	3.7 × 10^−8^	0.06	4.3 × 10^−8^	0.01	0.8	1.0	0.01
2	2:60679942	60679942	C/T	4.5 × 10^−6^	0.5 ± 1.18	3.7 × 10^−5^	0.5 ± 1.2	2.1 × 10^−5^	0.01	0.0002	0.04	0.9	0.9	0.9
2	2:60748758	60748758	G/A	5.07 × 10^−6^	0.5 ± 1.14	5.0 × 10^−6^	0.6 ± 1.1	9.1 × 10^−6^	0.43	3.5 × 10^−6^	0.9	0.6	1.0	0.6
2	2:60696528	60696528	C/T	1.6 × 10^−5^	2.2 ± 1.2	0.06	2.4 ± 1.6	1.2 × 10^−6^	0.02	2.2 × 10^−6^	0.82	0.8	1.0	0.3

## Data Availability

The data that support the findings of this study are available from the corresponding author (M.B.) upon reasonable request.

## Supplementary Materials

The following supporting information can be downloaded at: https://www.mdpi.com/article/10.3390/genes15040469/s1, Supplementary Figure S1: Overall quality of targeted sequence data: aggregated report from MultiQC of all the FastQC reports of the sequence alignment data of the 192 samples. FastQC report shows (A) Per Sequence Quality, (B) Mean Sequence Quality Scores, (C) Per Sequence GC Content, and (D) Sequence Duplication Levels; Supplementary Figure S2: PCA plot of the phenotypic groups Hemolytic, VOC, and less severe; Supplementary Figure S3: (A) *BCL11A* interaction with 43 genes either in physical, co-expression, or both pathway networks. (B) Main functions of the 43 genes that interact with *BCL11A*; Supplementary Figure S4. Partitioning heritability by functional annotation using genome-wide association summary statistics. (A) Plot of proportion of heritability partitioning by functional annotation and (B) heritability enriched by functional annotation. Supplementary Table S1A. Custom enrichment panel of targeting regions based on which 192 sickle cell patients from Angola were sequenced; Supplementary Table S1B. Count and proportion of pathogenic variants of the identified 26 mutant genes in 192 SCA Angolan samples; Supplementary Table S1C. Genes with high burdens of deleterious and loss-of-function mutations in 192 SCA patients from Angola; Supplementary Table S2. Data obtained from 1000 Genomes Project (1KGP) and the African Genome Variation Project (AGVP) and used for analysis; Supplementary Table S3. Genetics distance (Fst) based on 237,572 common variants from the study targeted sequence data among the 20 ethnolinguistic cultural groups and HbF Angola; Supplementary Table S4. Top nominal significant variants from (1) logistic association test of VOC against hemolytic and (2) logistic association test of Lower versus Higher HbF.
