# Supplementary material for: Genetic Modifiers of Sickle Cell Anemia Phenotype in a Cohort of Angolan Children
Source: Genes (Basel). 2024 Apr 8;15(4):469. doi: 10.3390/genes15040469 (PMC11049512; doi:10.3390/genes15040469)
Supplement: Supplementary file 1 [file genes-15-00469-s001.zip › Supplementary Figures.pdf]

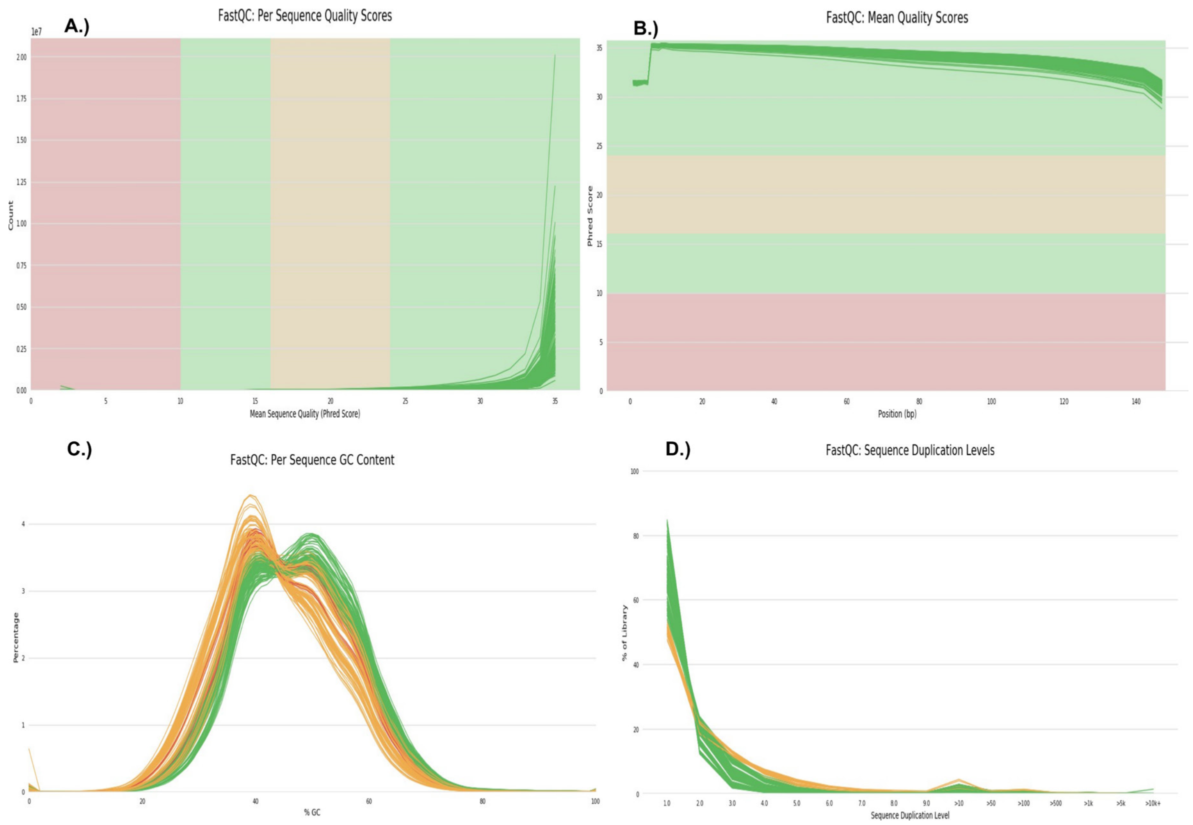

Figure S1

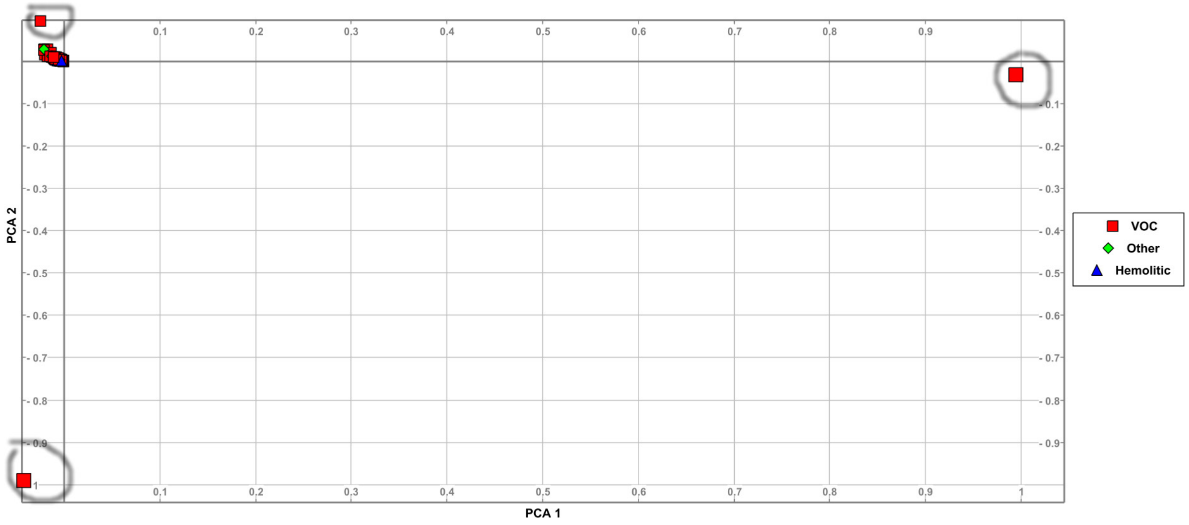

Figure S2

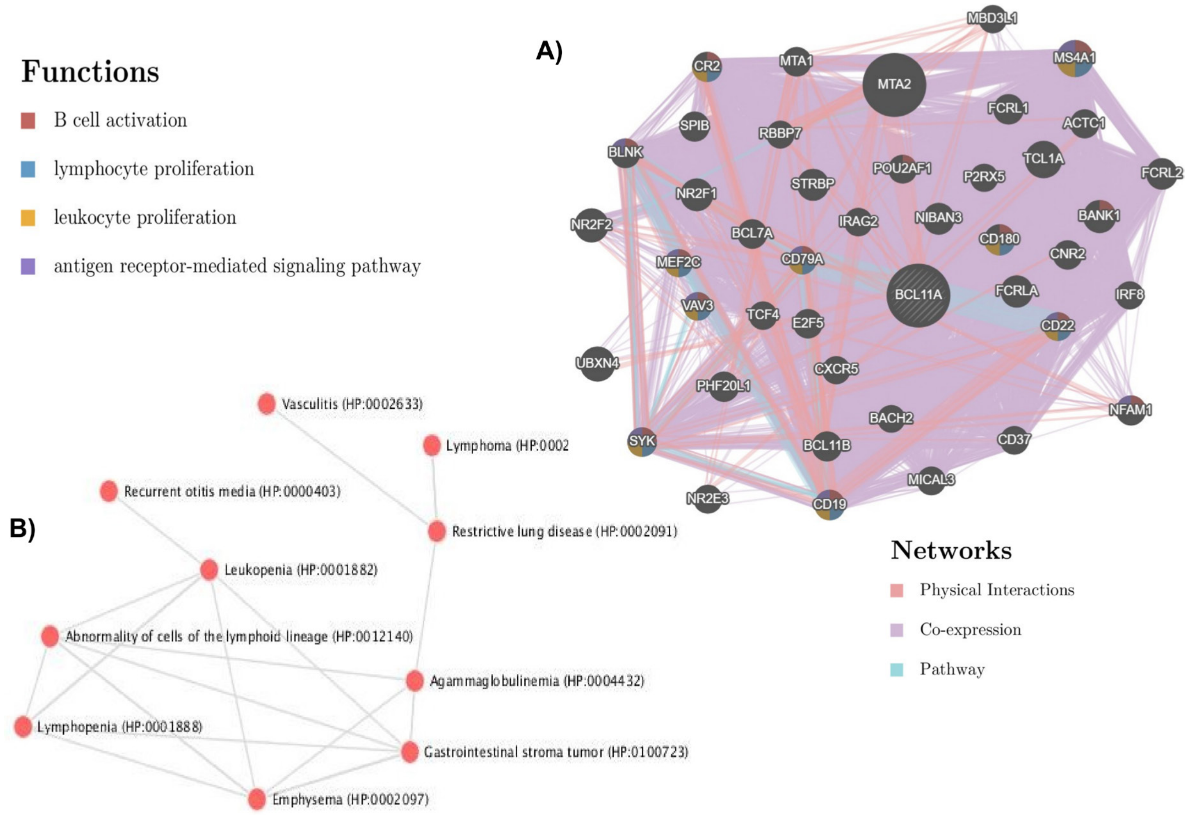
A)
Functions
B cell activation
lymphocyte proliferation
leukocyte proliferation
antigen receptor-mediated signaling pathway
MBD3L1
MS4A1
CR2
MTA1
MTA2
FCRL1
ACTC1
SPIB
RBBP7
BLNK
TCL1A
POU2AF1
P2RX5
FCRL2
NR2F1
STRBP
NR2F2
IRAG2
NIBAN3
BANK1
BCL7A
CD180
CD79A
CNR2
MEF2C
BCL11A
FCRLA
IRF8
VAV3
TCF4
E2F5
CD22
UBXN4
CXCR5
PHF20L1
NFAM1
BACH2
SYK
BCL11B
CD37
MICAL3
NR2E3
CD19
Networks
Physical Interactions
Co-expression
Pathway
B)
Vasculitis (HP:0002633)
Lymphoma (HP:0002
Recurrent otitis media (HP:0000403)
Restrictive lung disease (HP:0002091)
Leukopenia (HP:0001882)
Abnormality of cells of the lymphoid lineage (HP:0012140)
Agammaglobulinemia (HP:0004432)
Lymphopenia (HP:0001888)
Gastrointestinal stroma tumor (HP:0100723)
Emphysema (HP:0002097)

Figure S3

A.

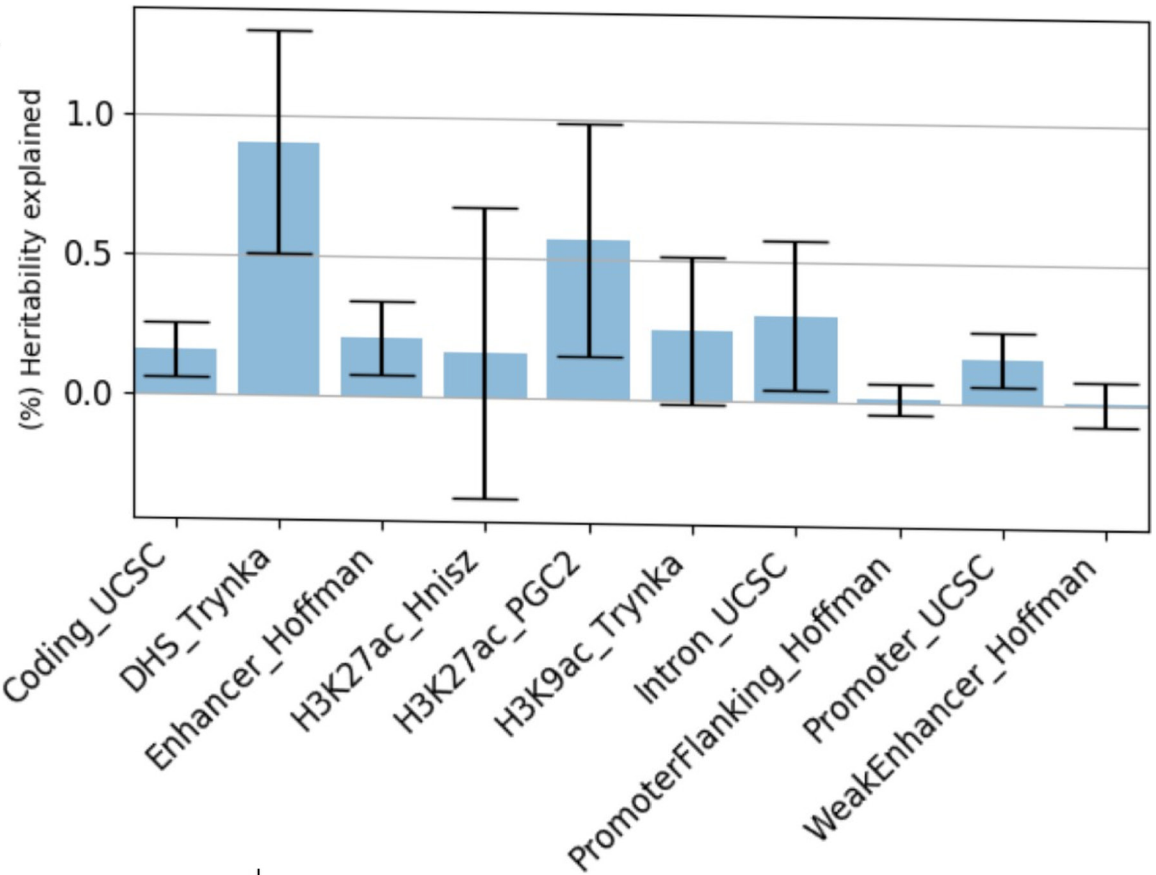

(%) Heritability explained
1.0
0.5
0.0
Coding_UCSC
DHS_Trynka
Enhancer_Hoffman
H3K27ac_Hnisz
H3K27ac_PGC2
H3K9ac_Trynka
Intron_UCSC
PromoterFlanking_Hoffman
Promoter_UCSC
WeakEnhancer_Hoffman

B.

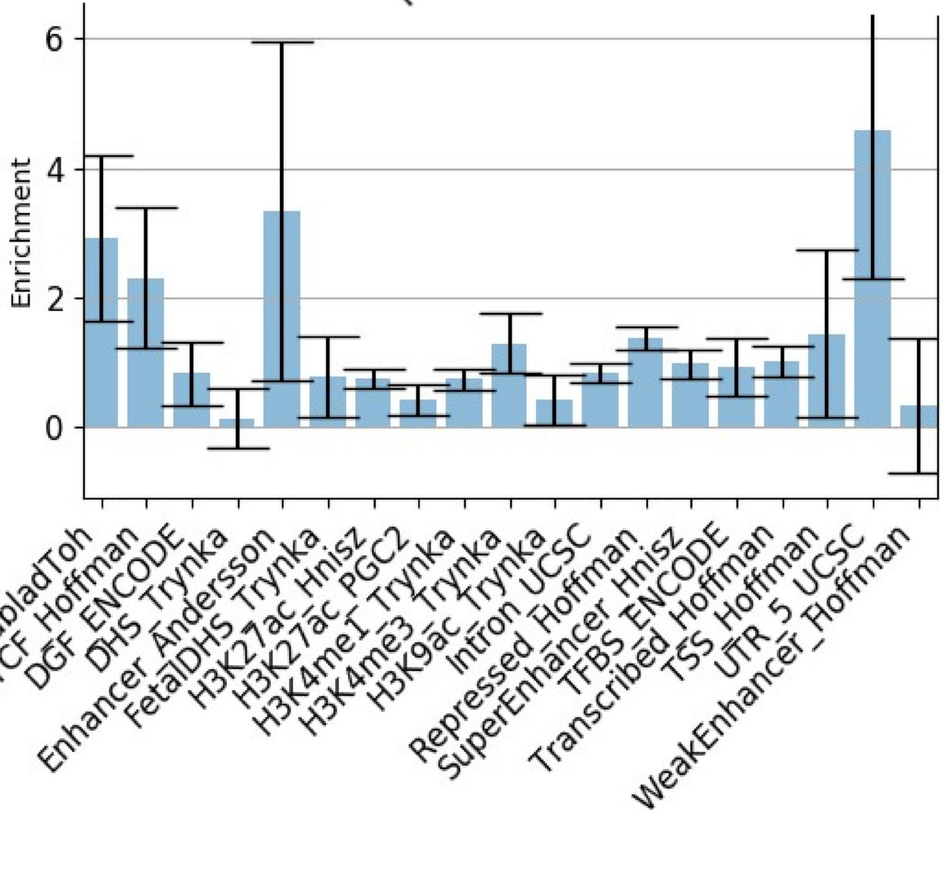

Enrichment
6
4
2
0
Conserved_LindbladToh
CTCF_Hoffman
DGF_ENCODE
DHS_Trynka
Enhancer_Andersson
FetalDHS_Trynka
H3K27ac_Hnisz
H3K27ac_PGC2
H3K4me1_Trynka
H3K4me3_Trynka
H3K9ac_Trynka
Intron_UCSC
Repressed_Hoffman
SuperEnhancer_Hnisz
TFBS_ENCODE
Transcribed_Hoffman
TSS_Hoffman
UTR_5_UCSC
WeakEnhancer_Hoffman

Figure S4
