# Supplementary material for: Genetic Modifiers of Sickle Cell Anemia Phenotype in a Cohort of Angolan Children
Source: Genes (Basel). 2024 Apr 8;15(4):469. doi: 10.3390/genes15040469 (PMC11049512; doi:10.3390/genes15040469)
Supplement: Supplementary file 1 [file genes-15-00469-s001.zip › Supplementary Table S1.pdf]

Supplementary Table S1A. Custom enrichment panel of targeting regions based on which 192 study sickle cell patients from Angola were sequenced.

Location	Chromosome	start	end
HBB cluster LCR 1	chr11	5225101	5275401
HBB cluster LCR 2	chr11	5275201	5325416
BCL11A 1	chr2	60678502	60729633
BCL11A 2	chr2	60729433	60780833
HMIP 2b intergenic HBS1L MYB 1	chr6	135281516	135367916
HMIP 2b intergenic HBS1L MYB 2	chr6	135367716	135454116
HMIP 2b intergenic HBS1L MYB 2	chr6	135452116	135540311
KLF1	chr19	12995036	12998217
Fox3 exon1b	chr6	108881018	108881238
Fox3 exon1	chr6	108882069	108883052
Fox3 exon2	chr6	108984638	108986122
Fox3 exon3	chr6	109001000	109006000
Fox3 exon1c	chr6	108977539	108977751
ZBTB7A exon1a CpG	chr19	4066660	4067442
ZBTB7A exon 1b	chr19	4065428	4065730
ZBTB7A exon 2	chr19	4053949	4055265
ZBTB7A exon 3	chr19	4044362	4048282
HBa1 HBa2	chr16	170847	178542
rs2342395	chr7	45975165	45975265
rs2965073	chr7	45981073	45981173
rs3111314	chr7	45981488	45981588
rs1525819	chr7	45984850	45984950
rs113143441	chr7	45987480	45987580
rs62456326	chr7	45987874	45987974
rs2462328	chr7	45992732	45992832
rs1722148	chr7	45993446	45993546
rs788766	chr7	45997493	45997593
rs788768	chr7	46000504	46000604
rs1404160	chr7	46014766	46014866

Supplementary Table S1B. Count and proportion of pathogenic variants of the identified 26 mutant genes in 192 SCA Angolan samples.

CHR	Gene	Band	#Pathogenic SNPs	#SNPs	% Pathogenic
6	*COL11A2*	p21.32	30	274	10,95
11	*DHCR7*	q13.4	11	181	6,08
7	*CLCN1*	q34	15	255	5,88
11	*LRP4*	p11.2	26	340	7,65
11	*PIDD1*	p15.5	22	68	32,35
7	*ABCF2*	q36.1	11	155	7,1
11	*TPP1*	p15.4	15	57	26,32
11	*DCHS1*	p15.4	22	336	6,55
2	*SNRNP200*	q11.2	10	171	5,85
11	*NDUFV1*	q13.2	10	53	18,87
6	*ITPR3*	p21.31	51	757	6,74
11	*ACAD8*	q25	10	161	6,21
7	*FLNC*	q32.1	29	255	11,37
7	*GIGYF1*	q22.1	15	157	9,55
7	*SLC4A2*	q36.1	23	144	15,97
6	*EHMT2*	p21.33	16	99	16,16
2	*ALPP*	q37.1	17	324	5,25
7	*PSPH*	p11.2	30	354	8,47
7	*PRSS1*	q34	103	1571	6,56
11	*PYGM*	q13.1	12	95	12,63
11	*ACCS*	p11.2	13	220	5,91
2	*CAD*	p23.3	14	179	7,82
11	*TCIRG1*	q13.2	13	79	16,46
7	*ABCB8*	q36.1	20	138	14,49
6	*NOTCH4*	p21.32	32	563	5,68
11	*NDUFS8*	q13.2	10	51	19,61

Supplementary Table S1C. Genes with high burdens of deleterious and loss-of-function mutations in 192 SCA patients from Angola.

CHROM	POS	A1/A2	Gene	Region	FuncGene	ExonicFunc
chr2	27446459	T/A	*CAD*	p23.3	exonic	nonsynonymous_SNV
chr2	27454368	G/A	*CAD*	p23.3	exonic	nonsynonymous_SNV
chr2	27454944	G/A	*CAD*	p23.3	exonic	nonsynonymous_SNV
chr2	27456571	G/A	*CAD*	p23.3	exonic	nonsynonymous_SNV
chr2	27458218	G/A	*CAD*	p23.3	exonic	nonsynonymous_SNV
chr2	27464801	T/A	*CAD*	p23.3	exonic	nonsynonymous_SNV
chr2	96949340	G/A	*SNRNP200*	q11.2	exonic	nonsynonymous_SNV
chr2	233245351	A/T	*ALPP*	q37.1	exonic	nonsynonymous_SNV
chr6	31848610	A/G	*EHMT2*	p21.33	exonic	nonsynonymous_SNV
chr6	32166740	G/A	*NOTCH4*	p21.32	exonic	nonsynonymous_SNV
chr6	32184972	A/T	*NOTCH4*	p21.32	exonic	nonsynonymous_SNV
chr6	33136301	T/C	*COL11A2*	p21.32	exonic	nonsynonymous_SNV
chr6	33626532	T/C	*ITPR3*	p21.31	exonic	nonsynonymous_SNV
chr6	33627323	A/T	*ITPR3*	p21.31	exonic	nonsynonymous_SNV
chr6	33635033	T/C	*ITPR3*	p21.31	exonic	nonsynonymous_SNV
chr6	33643517	C/A	*ITPR3*	p21.31	exonic	nonsynonymous_SNV
chr7	56087374	C/T	*PSPH*	p11.2	exonic	nonsynonymous_SNV
chr7	100282107	G/A	*GIGYF1*	q22.1	exonic	nonsynonymous_SNV
chr7	128483007	A/G	*FLNC*	q32.1	exonic	nonsynonymous_SNV
chr7	128484831	G/T	*FLNC*	q32.1	exonic	nonsynonymous_SNV
chr7	128484859	G/C	*FLNC*	q32.1	exonic	nonsynonymous_SNV
chr7	128486137	C/T	*FLNC*	q32.1	exonic	nonsynonymous_SNV
chr7	128490971	T/A	*FLNC*	q32.1	exonic	nonsynonymous_SNV
chr7	128493883	A/C	*FLNC*	q32.1	exonic	nonsynonymous_SNV
chr7	142458511	G/T	*PRSS1*	q34	exonic	nonsynonymous_SNV
chr7	142458523	T/A	*PRSS1*	q34	exonic	nonsynonymous_SNV
chr7	142460339	G/A	*PRSS1*	q34	exonic	nonsynonymous_SNV
chr7	142460374	A/G	*PRSS1*	q34	exonic	nonsynonymous_SNV
chr7	143027877	G/A	*CLCN1*	q34	exonic	nonsynonymous_SNV
chr7	150731652	G/T	*ABCB8*	q36.1	exonic	nonsynonymous_SNV
chr7	150738291	G/A	*ABCB8*	q36.1	exonic	nonsynonymous_SNV
chr7	150768572	C/T	*SLC4A2*	q36.1	exonic	nonsynonymous_SNV
chr7	150772851	T/C	*SLC4A2*	q36.1	exonic	nonsynonymous_SNV
chr7	150915925	T/G	*ABCF2*	q36.1	exonic	nonsynonymous_SNV
chr11	799869	G/A	*PIDD1*	p15.5	exonic	nonsynonymous_SNV
chr11	6636711	C/T	*TPP1*	p15.4	exonic	nonsynonymous_SNV
chr11	6661676	A/G,T	*DCHS1*	p15.4	DCHS1	exonic
chr11	44099444	A/T	*ACCS*	p11.2	exonic	nonsynonymous_SNV
chr11	46880727	C/T	*LRP4*	p11.2	exonic	nonsynonymous_SNV
chr11	46905461	A/T	*LRP4*	p11.2	exonic	nonsynonymous_SNV
chr11	46920139	A/G	*LRP4*	p11.2	exonic	nonsynonymous_SNV
chr11	46921459	A/G	*LRP4*	p11.2	exonic	nonsynonymous_SNV
chr11	64519479	G/A	*PYGM*	q13.1	exonic	nonsynonymous_SNV
chr11	64520657	A/T	*PYGM*	q13.1	exonic	nonsynonymous_SNV
chr11	64526116	T/G	*PYGM*	q13.1	exonic	nonsynonymous_SNV
chr11	67379861	C/T	*NDUFV1*	q13.2	exonic	nonsynonymous_SNV
chr11	67803739	A/C	*NDUFS8*	q13.2	exonic	nonsynonymous_SNV
chr11	67812434	G/A	*TCIRG1*	q13.2	exonic	nonsynonymous_SNV
chr11	71148958	T/C	*DHCR7*	q13.4	exonic	nonsynonymous_SNV
chr11	134127145	T/G	*ACAD8*	q25	exonic	nonsynonymous_SNV
chr11	134128410	A/G	*ACAD8*	q25	exonic	nonsynonymous_SNV
chr11	134128969	T/A	*ACAD8*	q25	exonic	nonsynonymous_SNV
