# Supplementary material for: Genetic Modifiers of Sickle Cell Anemia Phenotype in a Cohort of Angolan Children
Source: Genes (Basel). 2024 Apr 8;15(4):469. doi: 10.3390/genes15040469 (PMC11049512; doi:10.3390/genes15040469)
Supplement: Supplementary file 1 [file genes-15-00469-s001.zip › Supplementary Table S2.pdf]

Supplementary Table S2. Data obtained from 1000 Genomes Project (1KGP) (Consortium et al., 2012) and the African Genome Variation Project (AGVP) (Gurdasani et al., 2015) and used for analysis.

Population label	Ethnic group	Population description	Total Samples
AFR	Afro-Asiatic Semitic	Amhara:Ethiopia	22
	African-American	Americans of African Ancestry in SW USA (ASW)	60
	African-Caribbean	African Caribbeans in Barbado (ACB)	96
	Afro-Asiatic	Al-Gharbiyah, NA, Monufia, Kafrel-Sheikh, Mansoura, Alexandria, Dakahlia, Samanoud, Al-Buhayrah, Minya, AlSharqia, El-Mahalla all from Egypt	99
	Afro-Asiatic Cushitic	Oromo, Somali from Ethiopia	47
	Afro-Asiatic Omotic	Wolayta from Ethiopia	24
	KhoeSan	Khoe-San:Khoesan	84
	Niger Congo Bantu	Baganda, Banyarwanda, Barundi, RwandeseU- gandan, Banyankole:Uganda Bakiga, Mutan- zania, Basoga, other uganda gwas unknown, Mutooro, Batooro, Nyanjiro (Tanzania) from Uganda and Luhya in Webuye, Kenya (LWK)	2158
	Niger-Congo Bantu South	Zulu	98
	Niger-Congo Volta Niger	Esan in Nigeria (ESN), Yoruba in Ibadan, Nigeria (YRI)	205
	Niger-Congo West	Gambian in Western Divisions in the Gambia (GWD), Mende in Sierra Leone (MSL)	198
AMR	Latin American	Puerto Ricans from Puerto Rico (PUR), Colombians from Medellin, Colombia (CLM), Peruvians from Lima, Peru (PEL), Mexican Ancestry from Los Angeles USA (MXL)	347
EUR	European Center	British in England and Scotland (GBR)	91
	European North	Finnish in Finland (FIN)	99
	European South	Iberian Population in Spain (IBS), Toscani in Italia (TSI)	214
	European USA	Utah Residents with Northern and Western European Ancestry (CEU)	99
EAS	East Asian	Southern Han Chinese (CHS), Chinese Dai in Xishuangbanna, China (CDX), Kinh in Ho Chi Minh City, Vietnam (KHV), Han Chinese in Beijing, China (CHB), Japanese in Tokyo, Japan (JPT)	504
SAS	South Asian	Punjabi from Lahore, Pakistan (PJL), Bengali from Bangladesh (BEB)	180
	UK Indian	Sri Lankan Tamil from the UK (STU), Indian Telugu from the UK (ITU)	204
	USA Indian	Gujarati Indian from Houston, Texas (GIH)	103
**Total**			4,932
